# Prediction of Dengue Incidence Using Search Query Surveillance

**DOI:** 10.1371/journal.pntd.0001258

**Published:** 2011-08-02

**Authors:** Benjamin M. Althouse, Yih Yng Ng, Derek A. T. Cummings

**Affiliations:** 1 Department of Epidemiology, Johns Hopkins Bloomberg School of Public Health, Baltimore, Maryland, United States of America; 2 Headquarters Medical Corps, Singapore Armed Forces, Singapore, Singapore; Centers for Disease Control and Prevention, United States of America

## Abstract

**Background:**

The use of internet search data has been demonstrated to be effective at predicting influenza incidence. This approach may be more successful for dengue which has large variation in annual incidence and a more distinctive clinical presentation and mode of transmission.

**Methods:**

We gathered freely-available dengue incidence data from Singapore (weekly incidence, 2004–2011) and Bangkok (monthly incidence, 2004–2011). Internet search data for the same period were downloaded from Google Insights for Search. Search terms were chosen to reflect three categories of dengue-related search: nomenclature, signs/symptoms, and treatment. We compared three models to predict incidence: a step-down linear regression, generalized boosted regression, and negative binomial regression. Logistic regression and Support Vector Machine (SVM) models were used to predict a binary outcome defined by whether dengue incidence exceeded a chosen threshold. Incidence prediction models were assessed using 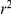 and Pearson correlation between predicted and observed dengue incidence. Logistic and SVM model performance were assessed by the area under the receiver operating characteristic curve. Models were validated using multiple cross-validation techniques.

**Results:**

The linear model selected by AIC step-down was found to be superior to other models considered. In Bangkok, the model has an 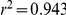, and a correlation of 0.869 between fitted and observed. In Singapore, the model has an 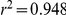, and a correlation of 0.931. In both Singapore and Bangkok, SVM models outperformed logistic regression in predicting periods of high incidence. The AUC for the SVM models using the 75th percentile cutoff is 0.906 in Singapore and 0.960 in Bangkok.

**Conclusions:**

Internet search terms predict incidence and periods of large incidence of dengue with high accuracy and may prove useful in areas with underdeveloped surveillance systems. The methods presented here use freely available data and analysis tools and can be readily adapted to other settings.

## Author Summary

Improvements in surveillance, prediction of outbreaks and the monitoring of the epidemiology of dengue virus in countries with underdeveloped surveillance systems are of great importance to ministries of health and other public health decision makers who are often constrained by budget or man-power. Google Flu Trends has proven successful in providing an early warning system for outbreaks of influenza weeks before case data are reported. We believe that there is greater potential for this technique for dengue, as the incidence of this pathogen can vary by a factor of ten in some settings, making prediction all the more important in public health planning. In this paper, we demonstrate the utility of Google search terms in predicting dengue incidence in Singapore and Bangkok, Thailand using several regression techniques. Incidence data were provided by the Singapore Ministry of Health and the Thailand Bureau of Epidemiology. We find our models predict incident cases well (correlation greater than 0.8) and periods of high incidence equally well (AUC greater than 0.95). All data and analysis code used in our study are available free online and can be adapted to other settings.

## Introduction

Google has reported success in using terms entered into its search engine (www.google.com) to predict trends in Influenza-Like Illness (ILI) cases one to two weeks ahead of the US CDC Morbidity and Mortality Weekly Report [1]. Several studies have reported similar results for influenza surveillance using Google search data, Yahoo search data, and internet advertising [2]–[8]. The Google research indicates that as the weekly incidence of influenza increases or decreases, the volume of certain internet search terms within the same geographical region change with a high level of correlation and predictability. Using a near real-time ability to collect search data (within 24 hours as opposed to one to two week lead time for US CDC reporting), the researchers were able to obtain information on the trend of ILI patterns in a more timely fashion than traditional surveillance.

Though the first efforts to use search terms from www.google.com have focused on influenza, this pathogen may be one of the more difficult to predict using internet searches. The presentation is non-specific to the pathogen and many searching behaviors that an ill person with influenza might exhibit overlap with searching by individuals afflicted by other pathogens. Pathogens exhibiting a distinct clinical presentation described by disease-specific terms that are widely used by the general population might exhibit the clearest correlation of search terms with disease incidence. Additionally, prediction of incidence is more important for pathogens that exhibit strong temporal variation. Dengue exhibits both of these characteristics: It exhibits a more distinct clinical presentation than influenza, giving rise to more disease-specific terms; and, dengue incidence in many locations exhibits large interannual variability with incidence varying by as much as a factor of ten from one year to the next [9].

The dengue virus is an arboviral illness of the family *Flaviviridae* and consists of 4 antigenically distinct serotypes. It is transmitted by the bite of an infected mosquito (*Aedes aegypti* poses the biggest threat to humans) and infection by one serotype does not confer life-long immunity to another serotype. The incubation period is typically 4–7 days and may present with undifferentiated fever, petechiae rash, nuchal headache, myalgia and arthralgia. The severe clinical manifestation, dengue hemorrhagic fever (DHF), is strongly associated with second infections and arises in around 3% of cases [10].

Prediction of outbreaks of dengue virus in countries with underdeveloped surveillance is of great importance to ministries of health and other public health decision makers who are often constrained by budget or man-power. The clinical presentation of dengue, although overlapping with other pathogens, is more specific to dengue than ILI is to influenza infection, and many of the search terms that individuals might search for when seeking information on dengue are specific to dengue (as opposed to terms such as ‘cold’). Thus, internet searches might exhibit stronger correlation with dengue incidence than influenza. Accurate predictions of dengue incidence might allow for more effective targeting of control measures such as vector control and preparation for surges in patients among hospitals, and may significantly increase the rapidity of dengue predictions in areas with less developed surveillance systems.

In Thailand, dengue has been a significant source of morbidity and mortality for over 70 years. DHF was first observed in Bangkok in 1949. The Thai Ministry of Public Health has conducted dengue surveillance since 1968. Incidence in Bangkok varies widely from year from 15,000 cases to over 175,000 cases annually. In Singapore, DHF was a significant cause of childhood mortality, in the 1960s, 1970s and 1980s, prompting vector control efforts that reduced the density of *Aedes* mosquito breeding sites and precipitated a decline in the incidence of DHF in the late 1980s and early 1990s [11]. However, from the late 1990s onwards, there has been a resurgence of dengue fever despite low levels of *Aedes* mosquito breeding, culminating in the largest observed epidemic in Singaporean history of over 14,000 cases in 2005. Although there is a trend towards an increase during the middle of the year, there is a wide variation of weekly incidence ranging from 32 to 713 cases during the 2004–2011 period.

The ability to accurately predict a rise in incidence would be a useful way to trigger a series of clinical interventions (deployment of medical teams, clearing of hospitial beds), and public health interventions (escalation in surveillance, public health education and pre-emptive source reduction measures) to reduce the transmission of dengue fever. Internet search term based surveillance could decrease delays associated with traditional surveillance systems and support under-developed systems.

## Methods

### Search Query Data

**Figure 1:**
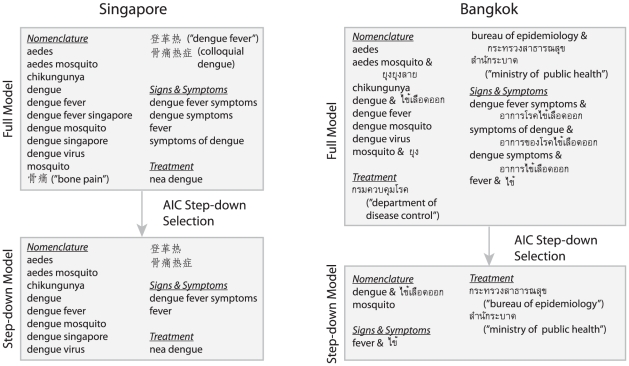
Schematic of step-down search term selection. Figure shows the search terms used in the full models for Singapore and Bangkok (top boxes), as well as the results of the AIC step-down procedure (bottom boxes).

Search data were downloaded from Google Insights for Search (http://www.google.com/insights/search/) on February 18th, 2011 for Singapore and March 2nd, 2011 for Bangkok. Relevant search terms for both were selected by brainstorming common words used in searching for dengue. We searched terms that include words in all three of the official languages in Singapore; English, Chinese Malay and Tamil. Terms for both Singapore and Bangkok were classified into 3 categories: nomenclature, signs/symptoms and treatment. The search terms for the “full models” are shown in Figure 1.

Google Insights for Search provides related searches that generate a significant volume of results. All relevant related search data were retrieved. Google Insights for Search ignores capitalization, but treats misspellings and different orderings (for example “symptoms flu” and “flu symptoms”) as distinct searches. However, the volume of search data for these are small and none were included in model testing.

Often, the Google Insight engine would only return data aggregated by month, because of uncertainty in weekly estimates in terms with low levels of search. For these terms a cubic spline was used to disaggregate the data to weekly responses (using R's spline()); negative values resulting from the spline were set to 0. The data were also regressed with the same model terms using the monthly aggregated data, and similar results were obtained (see below). Importantly, Google Insight returns a sample of the actual search volume, so exact replication of the estimates of the model covariates is impossible. To correct for seasonal variation and confounding by time we included both the month of the year (coded numerically as 1 for January, 2 for February, etc) and a numeric code indicating week and year of the current data point (given in R as the number of days since January 1st, 1970).

### Epidemiological Data

**Figure 2:**
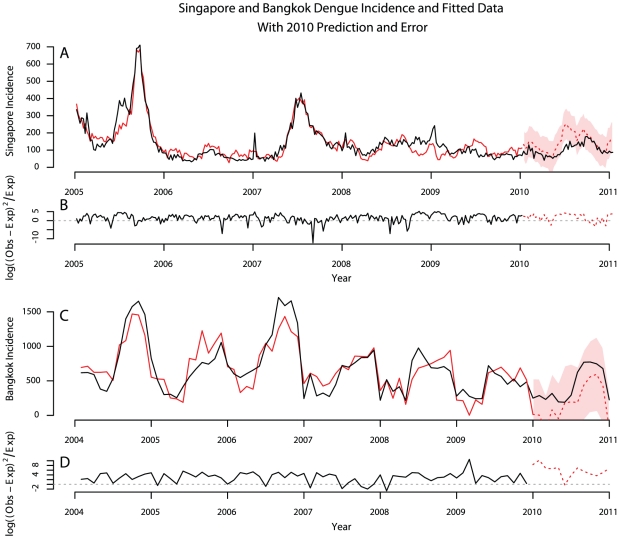
Correlation between observed dengue incidence and model fit. Figure displays observed dengue case incidence for years 2005–2011 (black lines) as well as the model fitted to data from 2005–2010 (solid red lines) and 2010 prediction with 95% prediction intervals (dashed red lines and solid red band) for both Singapore (panel A) and Bangkok data (panel C). Panels B and D show the error between the observed incidence and model fit or prediction.

Epidemiologic surveillance data were obtained from the Singapore Ministry of Health website which conducts routine epidemiological data collection via the government polyclinics, public hospitals, clinical laboratories as well as via mandatory communicable disease reporting procedures [11]. Clinical and laboratory confirmed dengue fever cases have been reported to the Ministry of Health since 1977 and the data are aggregated by week. Thai monthly incidence data were gathered from the Thai Bureau of Epidemiology website. Since Google provides data on internet searches only since 2004, we only considered dengue incidence data from 2004. Incidence data for both Singapore and Bangkok are presented as the black lines in Figure 2.

### Model Selection & Validation

We considered two outcomes, incident dengue cases and a binary outcome defined to be 1 during periods of high incidence and 0 otherwise. Multiple linear regression, negative binomial regression and generalized boosted regression (GBR) were used to model the weekly incidence of dengue fever using internet search terms [12]. A backwards and forwards step procedure was used to find the linear regression model that maximizes the Akaike information criterion (AIC). Negative binomial regression fit with the full set of search terms in each location was chosen over Poisson regression due to over-dispersion of the search term data. GBR models were fit using the gbm package in R [13].

Candidate models were trained using 2005–2010 data and used to predict 2011 incidence. Inclusion of 2004 data from Singapore reduced the predictive accuracy of the model. Because the predictions were not qualitatively different, and included a nearly overlapping set of covariates when 2004 was or was not included, we chose to optimize our predictions of incidence in later years by dropping 2004 from the models. To choose between the multiple linear regression, negative binomial regression and GBR models, we determined the model with the largest correlation between the 2010 prediction and lagged incidence. This model was then cross-validated to evaluate prediction performance. We used leave one out cross-validation and an expanding prediction window (both weekly and yearly, forward and backward) for search data dated between January 2005 and through December 2010, and evaluated the normalized root mean square error (NRMSE) of the predicted values of the left-out data from the observed incidence.

In addition to models predicting incidence, logistic regression and Support Vector Machine (SVM) models were used to predict periods of high incidence [12]. We built models for three different high incidence thresholds defined as the 50th, 75th and 90th percentiles of numbers of cases over the period 2005–2011. Model performance was assessed using the area under the receiver operating characteristic curve (AUC) for leave-one-out prediction.

All statistical analyses were conducted in R version 2.12.2 (R Core Development Team).

## Results

### Models to Predict Numbers of Incident Cases

The AIC step-down model outperformed the GBR and negative binomial model for predicting numbers of incident cases and was chosen as optimal in both Singapore and Bangkok. The best fitting AIC step-down models have the predictor search terms shown in Figure 1. Table 1 shows the model diagnostics comparing the step-down and full models for Singapore and Bangkok. A multiple time series plot showing normalized dengue incidence, the results of the optimized model fits and the error between predicted and observed incidence is presented as Figure 2.

**Table 1:** Incidence prediction model diagnostics.

		Singapore		Bangkok	
		Full	Step-down	Full	Step-down
	Terms	20	16	21	8
Model	*r* ^2^	0.948	0.948	0.947	0.943
Fit	Correlation	0.931	0.931	0.879	0.869
	AIC	2760.57	2751.559	999.162	986.712
Incidence	Lag-0 Correlation	–	0.666	–	0.921
Prediction	Lag-4 Correlation	–	0.785	–	0.762

Table reports the *r* ^2^, the correlation between observed dengue incidence and model fitted values, and the Akaike information criterion (AIC) for the full and step-down models in both Singapore and Bangkok. Table also reports correlation between observed 2010 dengue incidence and out-of-sample 2010 predictions from the step-down models for Singapore and Bangkok for unlagged observed incidence and observed incidence lagged by 4 weeks.

To assess the performance of the prediction on data that was not used to fit the model, we used multiple cross-validation techniques. We predicted incidence in 2010 in both locations using models fit to data from 2005–2010. Correlation between predictions of dengue in 2010 and observed dengue incidence for both Singapore and Bangkok are reported in Table 1.

We also assessed predictions for single and multiple observations that were left out of the data set used to fit the model. These results (reported in the Supporting Information S1) indicate a good fit of the step-down model relative to the full model. Additionally, the prediction errors are low in the leave-one-week-out case and the leave-52-weeks-out case. We also see poor performance of the negative binomial model relative to the other models.

### Models to Predict Periods of High Incidence

**Figure 3:**
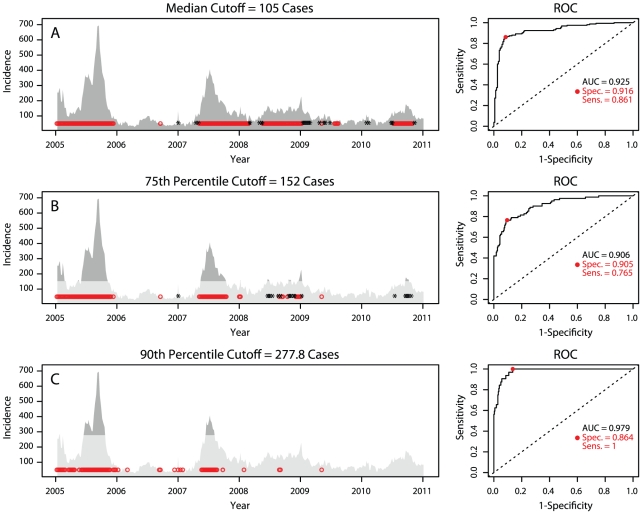
Summary of SVM prediction in Singapore. The performance of the SVM model in Singapore. Red circles indicate a prediction of high incidence at the optimal probability found from the ROC curve at right. Black stars indicate observed high incidence not predicted by the model. Panel A and the corresponding ROC curve at right indicate the median cutoff, panel B the 75th percentile cutoff and panel C the 90th percentile cutoff.

For both Singapore and Bangkok, logistic regressions and SVM models were fit to predict the binary outcome of incidence above or below a threshold. Figure 3 summarizes the prediction of the SVM model in Singapore (a similar graph presenting the Bangkok SVM model is presented in the Supporting Information S1), and Table 2 presents the AUC and optimal sensitivities and specificities for the logistic and SVM models for each of the three cutoffs. We can see good prediction for the median and 75th percentile cutoffs.

**Table 2:** Threshold prediction model diagnostics.

	Singapore			Bangkok		
Cutoff						
Percentile	50th	75th	90th	50th	75th	90th
No. cases	105	152	277.8	607	770.75	1134
SVM AUC	0.925	0.906	0.979	0.940	0.960	0.988
SVM Sens.	0.861	0.765	1.000	0.952	1.000	1.000
SVM Spec.	0.916	0.905	0.864	0.829	0.839	0.986

Table reports the AUC and optimal sensitivities and specificities for the LOO predictions for the SVM models at three threshold levels: the 50th, 75th, and 90th percentiles of dengue cases from 2005-2011 for Singapore and Bangkok, respectively.

We compared the performance of our model to a lag-1 autoregressive model using only dengue surveillance data from the last week (Singapore) or month (Bangkok) to predict the next observation. In Singapore, this model performs well, yielding a correlation between predictions and observed cases of 0.950. In Bangkok, the model performs much worse than models using search terms with a correlation of 0.766 (for comparison, the 8 search term model above has a correlation of 0.943). However, delays in compilation of these reports, especially in other locations could mean that these data would be unavailable for an autoregressive prediction model.

## Discussion

We have found that specific internet search terms are highly correlated with dengue incidence. Our best model for data from Singapore which included 16 terms showed a correlation of 0.931 with observed dengue incidence and an 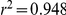. The 8 term model for Bangkok performs equally well with a correlation of 0.869 and an 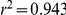. Out-of-sample predictions are predictably lower, but not significantly so. Our predictions of time periods with high dengue incidence are very accurate with sensitivities and specificities of 0.861–1.00 and 0.765–1.00 for multiple thresholds in each location. Together, these results demonstrate the viability of this data stream in supporting dengue surveillance.

Our model performed similarly to models built in other efforts to predict influenza incidence using internet search terms. Ginsberg et al. found a correlation of 0.90 for influenza incidence in the US using a model that included 45 search terms [1], and Polgreen et al. fit a series of models to influenza data in the United States and all had values of 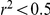 [4]. In out-of-sample prediction, our models performed slightly worse than the models of influenza produced by Ginsberg et al, which found a correlation of 0.97 (compared to 0.921 in Bangkok and 0.785 in Singapore). It should be noted that our model produces predictions for the entire year including high and low incidence seasons, whereas the models of Ginsberg produce predictions for only the influenza season. The accuracy of our predictions may be due to the clear clinical presentation of severe dengue. The larger interannual variability may also allow us to disentangle seasonal search behavior from dengue specific search behavior.

The search terms included in the models include nomenclature terms, terms describing signs and symptoms as well as treatment seeking. Interestingly, 11 of the 13 search terms that were found to be significant in our final model for Singapore were in English. This suggests that the typical language used for health seeking behavior in Singapore is English. In Bangkok, we also found that three of the seven significant terms are English.

We validated the candidate models using leave-one-observation-out, leave-one-year-out and forward and backward validation techniques. The model performance was fairly consistent across these different approaches. In our validations, we found one year with large incidence to be highly influential for the performance of our model (see Supporting Information S1). We expect that including future years with large incidence might further improve our results.

Singapore has an extremely well developed dengue surveillance system that makes reported cases available to policy makers and the general public with a delay of around one week. In a setting with this rapidity of reporting, it is challenging for an internet search term model to return results more quickly and with better performance than a model that uses only reported cases to predict future cases [14]. This point has been demonstrated elsewhere for predicting consumer behavior: predictive search term-based models perform better when used in conjunction with rich independent data sets [15]. Thus, in Singapore, this tool might best be used as a supplement to existing surveillance systems. However, in other settings, with less developed surveillance systems, an internet search term-based system may yield significant gains in the rapidity of predictions. In Thailand, there are significant delays in the reporting of cases from many areas of the country. Our model may give significant improvements in settings with significant delays. It is conceivable that some dengue-endemic settings in South and Southeast Asia may have significant internet use before surveillance systems are developed and thus an internet search term-based model may be a proxy for routine surveillance in these settings.

Caution must be used when generalizing our method to other settings. Even though we have chosen two settings that have very different rates of internet usage, both countries are of higher income than many of the countries in the region. However, it is reasonable to assume increasing internet penetration in the future. Individual models need to be developed for specific settings using local surveillance data and search terms. This effort shows that this approach may have promise in other settings.

There are several other limitations to our work. Internet searching behavior is susceptible to the impact of media reports as has been found for influenza systems [1], [16]. The rate of internet use and the rate of health information seeking in this setting may be changing over time and thus our parameters might need to shift over time to incorporate the impact of these changes. Although not affecting performance here, future outbreaks of other clinically similar diseases such as chikungunya may challenge the performance of our model for dengue. Finally, the Google Insight tool returns a sample of actual search data and limits the availability of search terms for which there are very few returns, often aggregating these terms to a large temporal discretization. This limits the utility of these terms for the purposes of prediction.

Search query surveillance is rapidly expanding into many areas of public health including the surveillance of noninfectious diseases and to influencing policy domains [17]–[21]. The current work demonstrates the utility of using search query surveillance to forecast the incidence of a tropical infectious disease. Additionally, and importantly, we have constructed forecasting models using freely available search query data from Google Insights and publicly available surveillance data from Singapore and Bangkok. In addition, we have developed these models using open source software from the R statistical project. Our approach can be readily adapted to other settings where other proprietary efforts can not be implemented. The approach may be an important tool in many dengue endemic settings in supporting the public health response to dengue.

## Supporting Information

Supporting Information S1 (PDF)
