# Supplementary material for: Prediction of Dengue Incidence Using Search Query Surveillance
Source: PLoS Negl Trop Dis. 2011 Aug 2;5(8):e1258. doi: 10.1371/journal.pntd.0001258 (PMC3149016; doi:10.1371/journal.pntd.0001258)
Supplement: Supporting Information S1 — (PDF) [file pntd.0001258.s001.pdf]

Supporting Information for Prediction of Dengue Incidence using Search Query Surveillance

Benjamin M. Althouse[1,*], Yih Yng Ng[2], Derek A.T. Cummings[1]
1 Department of Epidemiology, Johns Hopkins Bloomberg School of Public Health, Baltimore, Maryland, USA
2 Headquarters Medical Corps, Singapore Armed Forces, Singapore
∗ Corresponding author: Benjamin Althouse Email: balthous@jhsph.edu

Other Search Terms Considered

To account for potential biases in search terms, we assessed the inclusion of terms to correct for internet searches related to a popular music group named "dengue fever" including the word "band" and the lead singer's name, however, the search volume was too low to return results in both Singapore and Bangkok.

Incidence Prediction Model Selection and Validation

To choose between the full regression model, the AIC step-down model, and the generalized boosted regression model, we calculated the Pearson correlation between predicted and observed dengue incidence for 2010 for various weekly lags. Figure 1 shows the correlation between the predicted incidence and observed incidence at various time lags for the three candidate models in Singapore and Bangkok.

To assess the performance of the incidence prediction models we used multiple cross-validation techniques on data that was not used to fit the model. We performed leave-one-week-out (LOO), leave-52-weeks-out (L52O) validation, as well as "forward" and "backward" validation; the model was fit sequentially by adding a week starting in 2005 and going forward, and starting in 2011 going backward. Table 1 shows the percent normalized root mean square error (NRMSE) for the step-down AIC selected linear regression, the full multiple linear regression, and the negative binomial regression model for each of the cross-validation procedures. Figure 2 shows the NRMSE over time for the LOO and L52O validations of the step-down model, as well as the predicted values for the week or year left out.

Figure 3 shows the outcome on prediction in Singapore of fitting the model sequentially adding a year. We can see that prediction substantially improves after including the large epidemics in 2005 and 2007. This demonstrates the importance of including large epidemic peaks when training the model and suggests that future observed epidemics will function to improve model fit.

Figure 4 shows the results of the SVM model for predicting periods of high dengue incidence in Bangkok for the three thresholds.

Periods of High Incidence Prediction Model Selection

Table 2 shows, for each of the three thresholds, the AUCs, sensitivities and specificities of the SVM and logistic regression models for predicting periods of high incidence in both Singapore and Bangkok.

Sensitivity to Splining Google Insight Data

We assessed the sensitivity of our incidence prediction model results to the fact that splining had to be used to expand Google Insight's monthly reported data by estimating a model that included the Singapore weekly optimized model terms with the aggregated monthly data and obtained an $r^2 = 0.929$, and an of AIC = 822.779. We also ran an AIC step-down on the aggregated monthly data. The terms "dengue", "dengue virus", "fever", "登革热", "骨痛热症" and the month of the year were dropped from the model and the terms "dengue symptoms", "dengue fever singapore" and "mosquito" were added to the model when comparing the included terms with the model estimated for the weekly data.

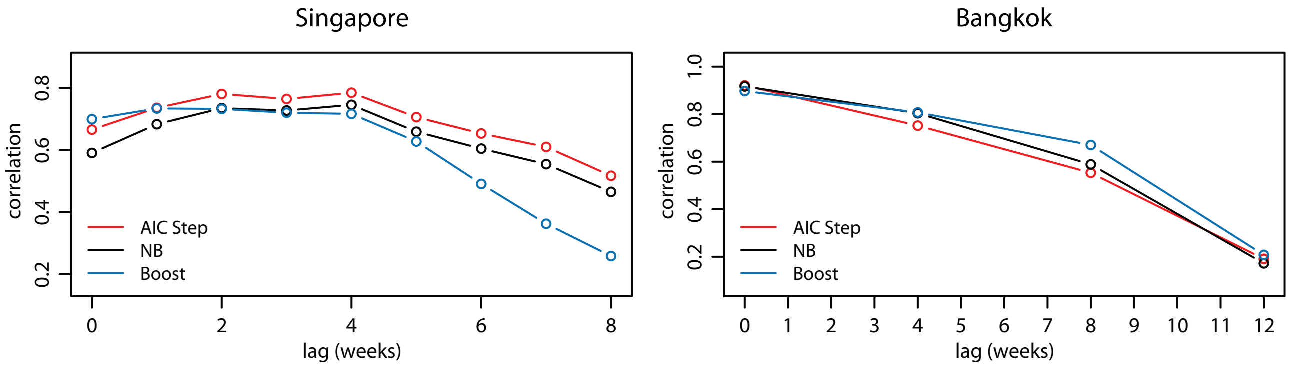

Figure 1: **Prediction Lag Correlation** Figure showing the correlation between the predicted incidence in Singapore from the two candidate models (AIC step-down and generalized boosted regression) and the observed incidence lagged from 0 to 8 weeks.

Singapore	AIC Step-down	Full	Negative Binomial
LOO	7.57% (IQR: 7.51, 7.63)	7.52% (IQR: 7.46, 7.59)	9.71% (IQR: 9.68, 9.75)
52-out	7.83% (IQR: 7.63, 8.14)	8.3% (IQR: 7.75, 9.22)	11.08% (IQR: 10.09, 13.73)
Forward	8.83% (IQR: 8.57, 20.17)	21.31% (IQR: 7.3, 33.33)	17.23% (IQR: 16.86, 20.75)
Backward	30.65% (IQR: 27.01, 39.72)	32.92% (IQR: 27.19, 38.97)	21.12% (IQR: 19.18, 22.46)

Bangkok	AIC Step-down	Full	Negative Binomial
LOO	14.65% (IQR: 13.55, 16.64)	14.66% (IQR: 13.47, 16.66)	14.75% (IQR: 13.2, 17.4)
52-out	12.74% (IQR: 12.6, 14.69)	12.32% (IQR: 12.26, 15.57)	18.35% (IQR: 17.53, 22.75)
Forward	14.08% (IQR: 12.86, 43.99)	15.78% (IQR: 12.75, 59.67)	22.74% (IQR: 17.21, 28.07)
Backward	38.02% (IQR: 31.94, 48.77)	53.01% (IQR: 36.06, 88.41)	35.95% (IQR: 31.82, 39.17)

Table 1: **Summary of Model Error** Table shows the percent normalized root mean square error for the step-down, full and negative binomial models for Singapore and Bangkok for a variety of cross-validation techniques. LOO indicates leave-one-week-out validation, "52-out" indicates leave-52-weeks-out validation, "Forward" indicates sequentially add one week starting in 2005, and "Backward" indicates sequentially add one week in reverse from 2011 to 2005.

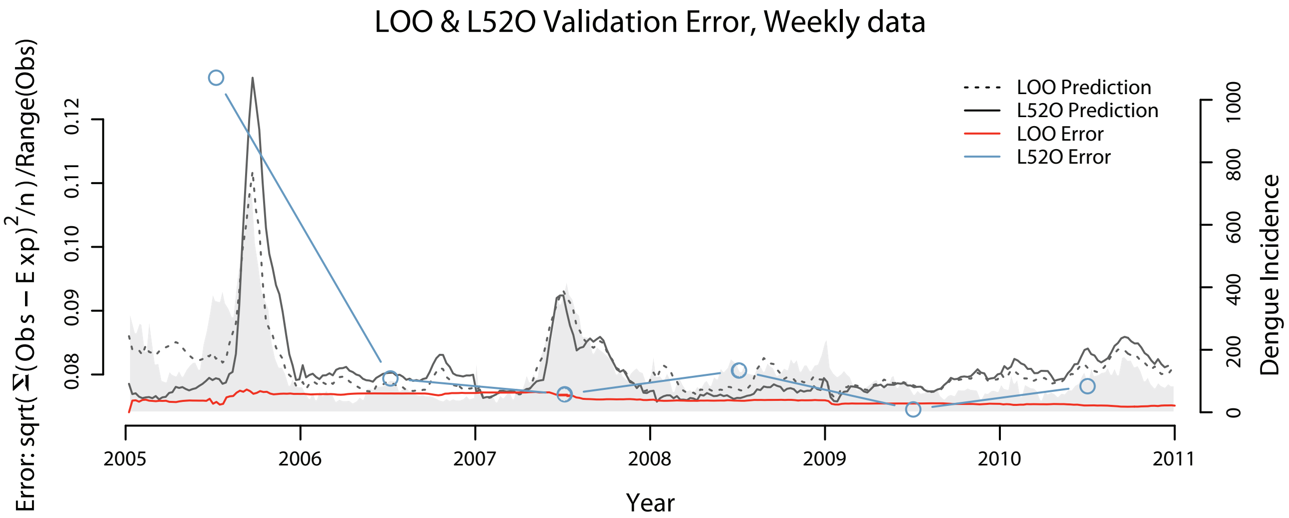

Figure 2: **Summary of Model Validation** This figure shows the results of the leave-one-week-out NRMSE (solid red line), the leave-52-weeks-out NRMSE (solid blue line) and the observed dengue fever case incidence (shaded grey region). The grey dotted line is the predicted incidence for the week left out and the grey solid line is the prediction for the 52 weeks left out.

	Singapore			Bangkok		
Cutoff Percentile	50th	75th	90th	50th	75th	90th
No. cases	105	152	277.8	607	770.75	1134
SVM AUC	0.925	0.906	0.979	0.940	0.960	0.988
SVM Sens.	0.861	0.765	1.000	0.952	1.000	1.000
SVM Spec.	0.916	0.905	0.864	0.829	0.839	0.986
Logistic AUC	0.922	0.896	0.922	0.917	0.960	0.988
Logistic Sens.	0.873	0.926	0.875	0.786	1.000	1.000
Logistic Spec.	0.844	0.675	0.954	0.951	0.839	0.986

Table 2: **Threshold Prediction Model Diagnostics** Table reports the AUC and optimal sensitivities and specificities for the leave-one-out predictions for the support vector machine (SVM) and logistic regression models at three threshold levels: the 50th, 75th, and 90th percentiles of dengue cases from 2005-2011 for Singapore and Bangkok respectively.

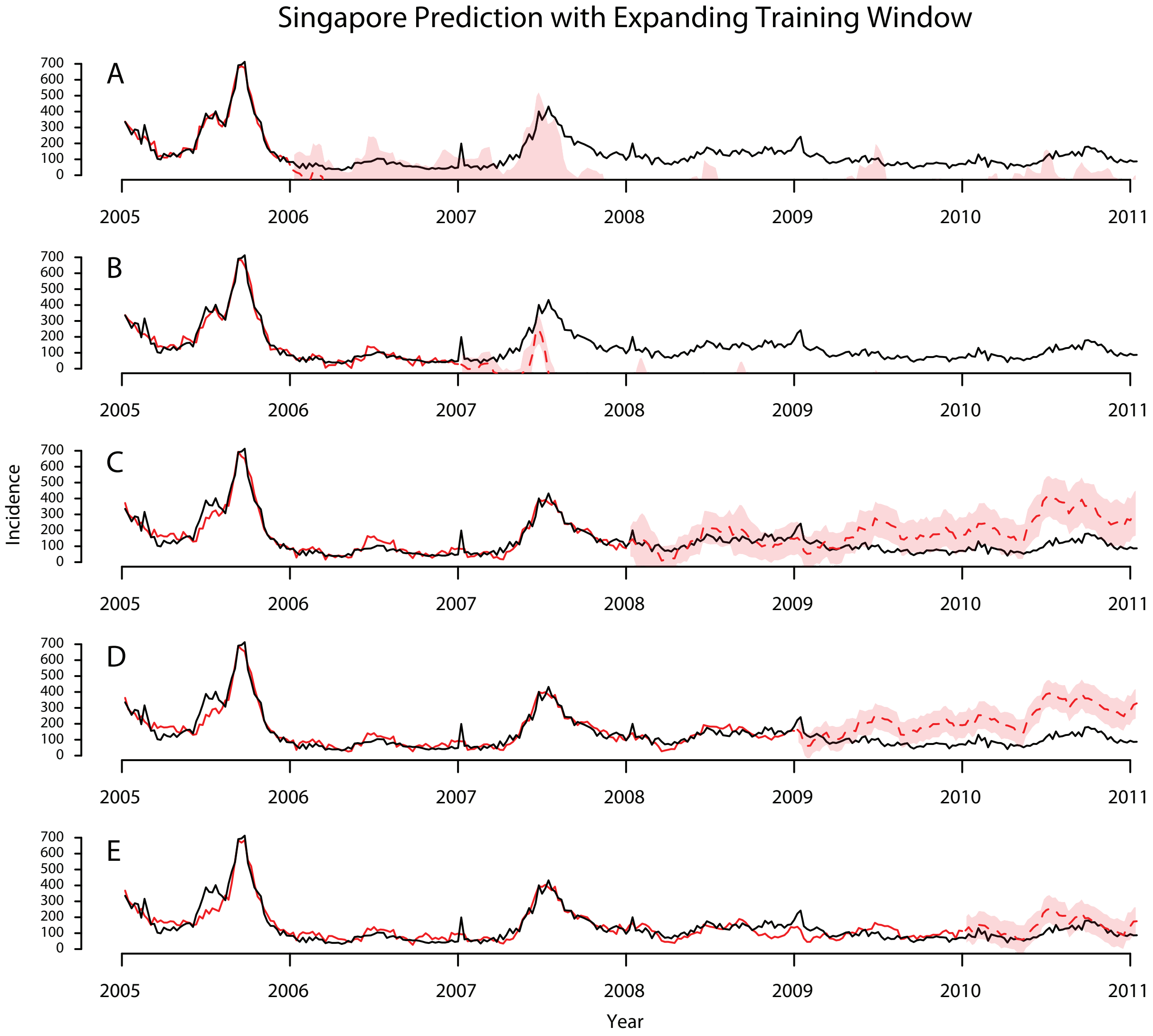

Figure 3: **Expanding Training Window** Figure showing the results of training the model with an expanding time window. Black lines indicate observed dengue case incidence, solid red lines indicate fitted model values, dashed red lines and red filled bands indicate predicted values and 95% prediction intervals, respectively. Panels (a), (b), (c), (d) and (e) show the model trained with 1, 2, 3, 3 and 5 years' worth of data, respectively.

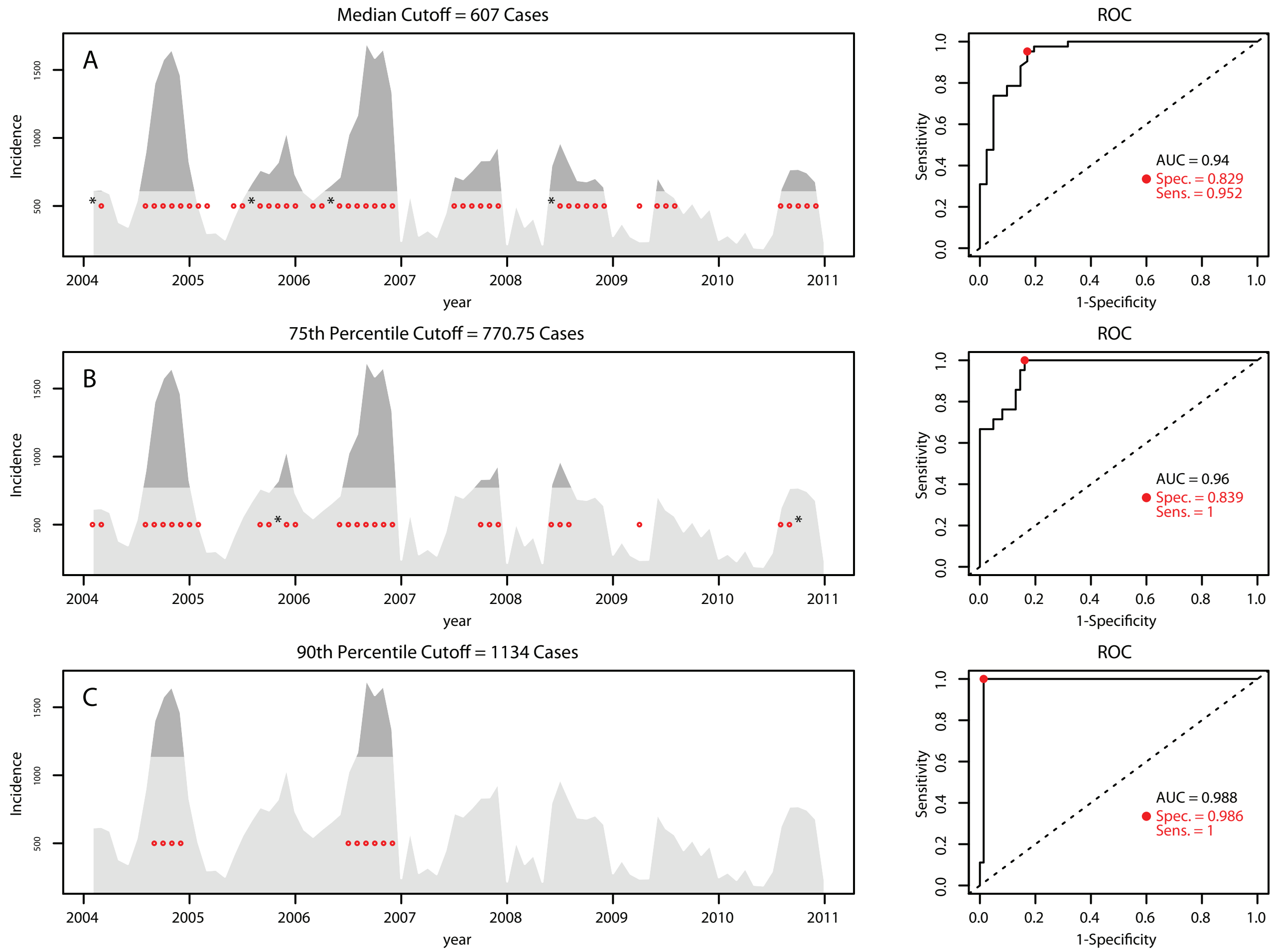

Figure 4: **Summary of SVM Prediction in Bangkok** The performance of the SVM model in Bangkok. Red circles indicate a prediction of high incidence at the optimal probability found from the ROC curve at right. Black stars indicate observed high incidence not predicted by the model. Panel A and the corresponding ROC curve at right indicate the median cutoff, panel B the 75th percentile cutoff and panel C the 90th percentile cutoff.
